# External ventricular drainage in pediatric patients: indications, management, and shunt conversion rates

**DOI:** 10.1007/s00381-024-06367-y

**Published:** 2024-04-01

**Authors:** Oday Atallah, Joachim K. Krauss, Elvis J. Hermann

**Affiliations:** Department of Neurosurgery, Hannover Medical School, Hannover, Germany

**Keywords:** External ventricular drainage, Children, Brain tumors, Intracranial hemorrhage, Infection

## Abstract

**Purpose:**

Placement of an external ventricular drainage (EVD) is one of the most frequent procedures in neurosurgery, but it has specific challenges and risks in the pediatric population. We here investigate the indications, management, and shunt conversion rates of an EVD.

**Methods:**

We retrospectively analyzed the data of a consecutive series of pediatric patients who had an EVD placement in the Department of Neurosurgery at Hannover Medical School over a 12-year period. A bundle approach was introduced to reduce infections. Patients were categorized according to the underlying pathology in three groups: tumor, hemorrhage, and infection.

**Results:**

A total of 126 patients were included in this study. Seventy-two were male, and 54 were female. The mean age at the time of EVD placement was 5.2 ± 5.0 years (range 0–17 years). The largest subgroup was the tumor group (*n* = 54, 42.9%), followed by the infection group (*n* = 47, 37.3%), including shunt infection (*n* = 36), infected Rickham reservoir (*n* = 4), and bacterial or viral cerebral infection (*n* = 7), and the hemorrhage group (*n* = 25, 19.8%). The overall complication rate was 19.8% (*n* = 25/126), and the total number of complications was 30. Complications during EVD placement were noted in 5/126 (4%) instances. Complications during drainage time were infection in 9.5% (12 patients), dysfunction in 7.1% (9 patients), and EVD dislocation in 3.2% (4 patients). The highest rate of complications was seen in the hemorrhage group. There were no long-term complications. Conversion rates into a permanent shunt system were 100% in previously shunt-dependent patients. Conversion rates were comparable in the tumor group (27.7%) and in the hemorrhage group (32.0%).

**Conclusion:**

EVD placement in children is an overall safe and effective option in children. In order to make further progress, carefully planned prospective and if possible randomized studies are needed controlling for multivariable aspects.

## Introduction

The placement of an external ventricular drainage (EVD) is a lifesaving intervention in acute hydrocephalus due to an intracranial hemorrhage or a tumor, but it is also an important part in the treatment of other disorders like infection of a shunt system or of a Rickham reservoir and other infections of the central nervous system causing hydrocephalus [1–4].

In a nationwide survey from Germany, in which data from 99 out of a total of 127 clinics were analyzed, the implantation of an EVD accounted for 5% of all neurosurgical procedures [5]. According to this survey, about 10,000 procedures are performed every year for the placement of an EVD embracing both children and adults [5]. Despite the efficacy of EVD insertion in a variety of intracranial pathologies, their benefits can be afflicted by procedural and catheter-related complications such as misplacement of the EVD, hemorrhage, along the catheter tract and EVD-related infection [4–12].

Despite the widespread use of EVD, there is still limited data available on its placement in pediatric patients both with regard to the underlying pathologies and the prevention of procedural and catheter-related complications [6, 8, 10, 11, 13–17]. In addition, the shunt conversion rates of an EVD into a permanent shunt system in pediatric patients have shown a wide variability [2, 18–22].

The goal of the present study was to investigate the indications, the management, and the complications of EVDs in a consecutive series of pediatric patients treated according to a uniform procedural concept and to determine the frequency and risk factors for shunt conversion.

## Material and methods

Demographic and clinical data from 126 pediatric patients treated in the Department of Neurosurgery at Hannover Medical School who had an EVD inserted between February 2008 and January 2020 were analyzed in this retrospective study. Clinical data which were collected included indications for EVD placement, number of EVDs placed, methods used for placement, duration of the EVD stay, procedural and catheter complications, and conversion rate into a permanent shunt system. Hannover Medical School is a tertiary care center with a catchment area of about 2 million inhabitants.

All patients with EVD placement from postnatal age until age 18 were included. Children transferred from other hospitals with an EVD already in place were excluded. Patients were categorized according to the underlying pathology that led to EVD placement: (1) tumor group—brain tumors with obstructive hydrocephalus requiring an EVD preoperatively or after tumor resection; (2) infection group—patients who required EVD placement, (a) after a previously placed shunt needed to be removed due to an infection, (b) after infected Rickham reservoir removal, or (c) with infections of the central nervous system causing hydrocephalus; and (3) hemorrhage group—hydrocephalus due to intracranial hemorrhage from various causes.

### Bundle approach

In all patients, a bundle approach was used which was implemented in 2006 in the Department of Neurosurgery at Hannover Medical School to standardize the placement and the care of EVDs for both in adults and in children [12] in order to minimize EVD-related infections.

The protocol for EVD placement included analgosedation, generous shaving of the hair at the insertion site, pre-procedural prophylactic intravenous antibiotics, strict antiseptic approach with nursing assistance, EVD tunneling (except when using a twist drill), a closed drainage EVD system, and occlusive wound dressings.

The EVD care protocol implemented strict antiseptic preventions before any EVD manipulation including hand disinfection, sterile gloving, scalp disinfection when changing dressings, and continued antibiotic prophylaxis during the whole drainage period. No routine CSF samples and no routine EVD replacements were performed.

### Techniques for EVD placement

#### Anatomical landmarks

In children older than 8 years, a standard frontal/pre-coronal burrhole on the right side was used. The burrhole was placed 2.5 cm lateral to the midline at Kocher’s point, and the tip of the catheter was positioned optimally just above the foramen of Monro using the external auditory canal and the ipsilateral epicanthus medialis as landmarks [23]. All EVDs were tunneled subcutaneously for a minimum of 5 cm and fixed at the skin by sutures.

#### Ultrasound guidance

In the first year of life, in case the anterior fontanelle was big enough and accessible providing an adequate window for ultrasound exposure, intraoperative ultrasound guidance was employed during EVD placement.

#### Electromagnetic navigation

Between the first year of life until age 8 and in children with distorted anatomy or more narrow ventricles, electromagnetic (EM) navigation was the preferred method for EVD placement [24–26].

EVDs from VentriGuard^®^, coated with silver (8.5 F), were used in all instances.

### Definition of EVD-related infection

EVD-related infection was defined according to the criteria of Lozier et al. as outlined previously [12, 27]. The diagnosis of an EVD-related infection required at least one of the criteria from a to d plus at least one criteria from e to g: (a) decreased CSF glucose level (< 40 mg/dl or < 50% of serum glucose level); (b) increased CSF protein level (> 50 mg/dl); (c) increased CSF cell count (> 15 cells/µl); (d). increased inflammatory factors in blood count (leucocytes > 10,200/µl, C-reactive protein (CRP) > 8 mg/l); (e) development of clinical presentation of meningitis (fever > 38.5 °C, nuchal rigidity, photophobia, reduced level of consciousness, seizures); (f) positive bacterial culture or gram staining in CSF sampling; and (g) radiological signs of ventriculitis in cerebral computed tomography (CT) or magnet resonance imaging (MRI).

All patients received Cephazoline i.v. adapted to body weight (50 mg/kg) before starting the surgery. Further, Cephazoline i.v. 3 times/day was administered prophylactically until removal of the EVD. In case of allergy to cephalosporins, clindamycin (25 mg/kg) was given.

In the case of an EVD-related infection during the drainage period, the EVD was replaced, and antibiotics were administered empirically. The antibiotic regime was adapted once a causative pathogen was determined.

Antibiotics after explantation of an infected shunt system were given at least for 2 weeks. Thereafter, CSF was screened for pathogenic microorganisms before a new shunt system was implanted.

### Statistics

Statistical analysis was carried out with SPSS. Categorical data were compared and tested for significance using Chi-square and Fisher’s exact test, while continuous data were tested for significance by Student’s *t*-test. The Mann–Whitney *U* Test was used for not normally distributed data. The Fisher-Freeman-Halton test was used to determine if there was a significant association between diagnoses and complications. Statistical significance was defined as a *p* value < 0.05.

## Results

Data from 126 patients were analyzed out of which 72 (57.1%) were male, and 54 (42.9%) were female. The mean age at the time of EVD placement was 5.2 ± 5.0 years (range 0–17 years).

### Indications for EVD placement

The majority of patients was assigned to the “tumor group” (*n* = 54, 42.9%). Patients either suffered from a tumor that lead to an obstructive hydrocephalus (*n* = 44) or required an EVD after tumor resection due to postoperative pneumocephalus (*n* = 6) or due to postoperative hydrocephalus (*n* = 4). Tumors of the posterior fossa accounted for the largest proportion in the tumor group (83.3%). During the study period, an EVD was placed prior to tumor resection if surgery was planned in the semi-sitting position, also in order to treat postoperative pneumocephalus.

The “infection group” consisted of 47 patients (37.3%) requiring an EVD after explantation of an infected shunt-system (*n* = 36), an infected Rickham reservoir (*n* = 4), or due to a bacterial or viral meningoencephalitis (*n* = 7).

The remaining 25 patients (19.8%) were assigned to the “hemorrhage group” requiring an EVD due to intracranial hemorrhage of various causes (3 aneurysms, 8 arteriovenous malformations, 3 tumor bleedings, and 6 intraparenchymal bleedings of unknown causes) or traumatic brain injury in 5 children.

The distribution of the various indications for EVD placement and their relative frequency with regard to the total cohort is shown in Table 1.

**Table 1:** Indications for external ventricular drainage placement in a pediatric cohort of 126 patients

Group	Indication for EVD placement	Cases	%
Tumor (*n* = 54)	Preoperative obstructive hydrocephalus	44	34.9
	Postoperative pneumocephalus	6	4.8
	Postoperative hydrocephalus	4	3.2
Infection (*n* = 47)	Shunt infection	36	28.6
	Rickham reservoir infection	4	3.2
	Meningoencephalitis	7	5.6
Hemorrhage (*n* = 25)	Intracerebral/intraventricular hemorrhage	20	15.8
	TBI	5	3.9

*EVD* external ventricular drainage, *TBI* traumatic brain injury

### Techniques used for EVD placement

In the total cohort of 126 patients, 107 EVDs were placed using anatomical landmarks, 9 using intraoperative ultrasound guidance, and 10 using EM navigation. The frequency of applying these different techniques according to the cause of hydrocephalus is outlined in Table 2.

**Table 2:** Techniques for external ventricular drainage placement in the different groups

Technique	Group						Total
	Tumor		Infection		Hemorrhage		
	Cases	%	Cases	%	Cases	%	
Anatomical landmarks	48	89%	40	85%	19	76%	**107**
Ultrasound guidance	2	4%	4	9%	3	12%	**9**
EM navigation	4	7%	3	6%	3	12%	**10**
**Total**	**54**	**100%**	**47**	**100%**	**25**	**100%**	**126**

*EVD* external ventricular drainage, *EM navigation* electromagnetic navigation

All EVDs were placed on the right side. In 66 patients (52.4%), the EVD was placed via a burrhole trepanation, in 23 (18.2%) using a twist drill, and in 37 (29.4%) via the old puncture canal of the explanted ventricular catheter of the infected shunt system. In 6 cases (4.8%), the EVD was inserted on the ICU using a twist drill, while in 120 cases (95.2%), it was implanted in the OR.

While the overall complication rate in this study was 19.8% (*n* = 25/126), the total number of complications was 30. Out of the 25 patients with complication, three had 2 complications, and one patient had 3 complications. The frequency of specific complications with regard to the three different groups is summarized in Table 3.

**Table 3:** Total number of complications of external ventricular drainage placement and drainage in relation to specific indications

Complications		Tumor group (*n* = 54)	Infection group (*n* = 47)	Hemorrhage group (*n* = 25)	Total (*n* = 126)	*p* value
**EVD placement**	Misplacement of the EVD	0	1	3	4 (3.2%)	***p* = 0.0121**
	Bleeding along the puncture canal	0	0	1	1 (0.8%)	***p* = 0.1984**
**During drainage**	Infection	5	3	4	12 (9.5%)	***p* = 0.4126**
	EVD dysfunction	1	3	5	9 (7.1%)	***p* = 0.0161**
	EVD dislocation	1	3	0	4 (3.2%)	***p* = 0.4152**
**Total**		**7 (13%)**	**10 (21.3%)**	**13 (52%)**	**30 (23.8%)**	

*EVD* external ventricular drainage

### Complications of EVD placement

During placement of the EVD, complications occurred in a total of 5/126 (4%) instances including misplacement of the EVD in 4 patients and an asymptomatic bleeding along the puncture canal in one patient. Misplacement was defined when the tip of the ventricular catheter was not located in the anterior horn of the ipsilateral ventricle. One misplacement was noted in the infection group using the old puncture canal of the explanted infected shunt catheter in a patient with narrow ventricles. Surgery was performed by a resident, and the catheter was repositioned subsequently with EM navigation. The other three misplacements occurred in the hemorrhage group. In one case with distorted anatomy and intraventricular hemorrhage, the EVD was misplaced despite using EM navigation by a staff neurosurgeon unexperienced in EM navigation, and the other two misplacements were secondary to free-hand EVD placement performed by a resident, respectively. All misplacements were asymptomatic. The EVD placement resulting in an asymptomatic bleeding along the puncture canal was performed also by a resident. No intervention was needed.

### Complications during drainage time of the EVD

**Fig. 1:**
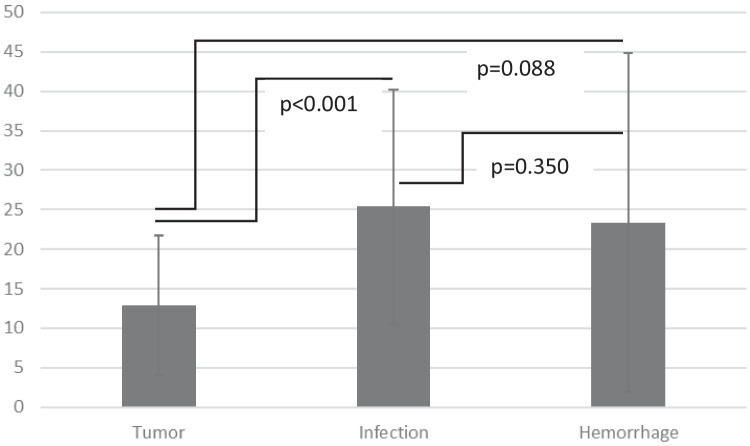
Duration of external ventricular drainage period in the three patient groups in days. *EVD *external ventricular drainage

The mean EVD drainage period was 19.5 ± 15.9 days (range, 1 to 85 days). There were significant differences in the EVD drainage time between the three patient groups (Fig. 1). The drainage period in the “tumor group” was significantly shorter than in the “infection group” (*p* < 0.001), while in the “hemorrhage group,” the drainage period was also shorter (*p* = 0.088).

Complications were observed in 25/126 patients (19.8%) including infection in 12 patients, EVD dysfunction in 9, and EVD dislocation in 4. In all 12 patients with an EVD-related infection, clinical signs of meningitis were observed, and a positive CSF culture was obtained. In three patients in the “infection group” a new microorganism was found during EVD drainage. The longest period of 85 days of EVD treatment occurred in the hemorrhage group in a 7-year-old child due to recurrent bleedings from a hypothalamic arteriovenous malformation causing dysfunction of the EVD three times.

Remarkably, however, no patient had sequelae subsequent to EVD complications on long-term follow-up.

### Spectrum of pathogenic microorganisms

Shunt infections were due to *Staphylococcus epidermidis*, *Staphylococcus aureus*, *Micrococcus luteus*, *Escherichia coli*, and *Klebsiella pneumoniae*.

Infected Rickham reservoirs were associated with *Staphylococcus aureus*, *Enterobacter cloacae*, and vancomycin-resistant Enterococcus. Primary infections (meningoencephalitis) were caused by *Candida parapsilosis*, *Pseudomonas aeroginosa*, *Staphylococcus aureus*, Epstein-Barr virus, and cytomegalovirus.

Various pathogens were identified as the cause of an EVD-related infection in 12 instances. The most common group of pathogens were Staphylococci (*n* = 8/12), and *Staphylococcus epidermidis* (*n* = 4/8) was the leading pathogen. Other pathogens included *Enterococcus faecium* (2/12), *Enterobacter aerogenes* (1/12), and *Escherichia coli* (1/12). The median duration of antibiotic treatment was 14 days (range, 10–20 days). The selection of the antibiotics used was based on the results of the antibiogram and after consultation with an infectologist. Table 4 shows the distribution of the pathogenic microorganisms with regard to the different EVD indications.

**Table 4:** Pathogenic microorganisms in external ventricular drainage-related infection

Pathogenic microorganisms	Groups			Total No. of cases (*n* = 126)
	Tumor (*n* = 54)	Infection (*n* = 47)	Hemorrhage (*n* = 25)	
	Cases	Cases	Cases	
*Staphylococcus aureus*	1	0	0	**1**
*Staphylococcus epidermidis*	2	1	1	**4**
*Staphylococcus capitis*	0	1	0	**1**
*Staphylococcus hominis*	0	0	1	**1**
*Staphylococcus cohnii*	0	1	0	**1**
*Enterococcus faecium*	1	0	1	**2**
*Enterobacter aerogenes*	0	0	1	**1**
*Escherichia coli*	1	0	0	**1**
**Total**	**5 (9.3%)**	**3 (6.4%)**	**4 (16%)**	**12 (9.5%)**

*EVD* external ventricular drainage

### Conversion rate to a shunt system

A total of 65 children (*n* = 65/126; 51.6%) required conversion of the EVD into a permanent shunt system. Significant differences in conversion rates were seen between the three patient groups. As expected, all patients in the infection group, who received an EVD after removal of an infected shunt system (*n* = 36), remained shunt-dependent. Out of the other 11 patients in the infection group, 10 patients needed a shunt system.

Patients in the other two groups had lower conversion rates: 15/54 (27.7%) in the “tumor group” and 8/25 (32%) in the “hemorrhage group.” There was no significant difference in the conversion rate between the “tumor group” and the “hemorrhage group” (*p* = 0.3067). Analyses within the “tumor group” showed that the conversion rate was slightly higher in patients who required the placement of an EVD before tumor resection (14/44 patients, 31.8%), than in those in whom the EVD was placed postoperatively due to pneumocephalus or hydrocephalus (1/10, 10%). However, the difference was statistically not significant (*p* = 0.252).

## Discussion

Our study demonstrates that EVD placement in pediatric patients is both efficacious and relatively safe. While our data are partially confirmatory regarding previously published series, they expand current knowledge since we did not restrict our analyses to certain pediatric populations but we included all consecutive cases over a longer period. These cross-sectional data show that, currently, the most frequent indication for EVD placement in children is related to brain tumors, followed by infection and hemorrhage.

The results of our study indicate that there are marked differences regarding the frequency of complications according to the cause for hydrocephalus requiring EVD placement. In particular, patients with hemorrhage are more prone to have misplacement of EVDs and to develop an infection during drainage or EVD dysfunction. The overall complication rate of EVD placement in children has been described to range between 3.4 and 32.2% [1, 17, 18, 28], but it has been difficult to appreciate exactly with regard to selection criteria for inclusion and exclusion of patients in study protocols and also the definition what constitutes an EVD-related complication. We will discuss some more specific aspects in the following. It is of outmost importance to have clear criteria to determine such disruptive interferes not only in hindsight when analyzing published data but also when planning new studies which require agreement in the pediatric neurosurgery community.

Highly variable rates for EVD misplacements and hemorrhages have been reported earlier [1, 9, 13, 14, 28, 29]. Anderson and colleagues noted a frequency of EVD misplacement of 8.8% in pediatric traumatic brain injury and a rate of asymptomatic hemorrhages of 17.6% [9], while Blaha et al. reported 10% of asymptomatic hemorrhages [13] and Stahl et al. 11.1% [29].

The rate for EVD misplacement and for bleeding along the puncture canal in our study was relatively low (overall 4%). We postulate that this is due to the use of EM-navigation when indicated. The usefulness of EM-navigation for EVD placement in reducing misplacement rates in children has been shown earlier [24–26]. In contrast to previous studies, we demonstrated the relevance of experience in EVD placement in children. When Kakarla et al. analyzed a series of 346 pediatric patients receiving an EVD, they reported a misplacement rate of 30% in children with traumatic brain injury, and they concluded that training experience had no input on the occurrence of EVD misplacement [30]. In our cohort, in 3/4 misplacements, the EVD had been placed by residents and in one instance by a surgeon on duty without ample expertise in EM-navigation. To avoid such complications, we now train every surgeon in the handling of EM-navigation for EVD placement on a regular basis. Our findings are in line with those of Dakson et al. who analyzed an adult cohort, stating that catheter misplacement was related to the level of training of the surgeon [31]. Furthermore, it would deserve consideration whether to place all ventricular catheters in children with navigation guidance.

Most published studies on pediatric EVD placement concerned EVD-related infections, reported to occur in up to 37% [32] and varying usually from 0.9 to 20% [16–18, 27, 32–37]. A major problem is the choice of criteria for definition of an EVD-related infection [12, 18, 27, 31, 32, 34, 37–41]. Most centers based on their experience suggest to use specific institutional guidelines for EVD management to reduce infection rates [16–18, 27, 32–36, 42]. We have described a multi-item bundle approach for EVD placement and care previously in a series of 208 patients which resulted in a reduction of EVD-related infection from 29.1% before its implementation to 4.8% thereafter. However, in this cohort, the majority of patients were adults, while only 11.2% belonged to the pediatric age group [12]. In our present pediatric cohort, the EVD-related infection rate was 9.5% indicating a higher vulnerability of children.

It appears that impregnation of EVD-catheters either with antibiotics or with silver particles may reduce related infections, but—for various reasons—this has not become standard yet [39, 43]. According to recent meta-analyses, infection rates were reduced from 13.7 to 3.6% using antimicrobial-impregnated EVDs [39]. When comparing the two different impregnated catheters in reducing infection rates, however, no significant difference was found. Both catheter types appear to have a higher impact on Gram-positive bacterial infection. On the other hand, the possibility of a selection of more aggressive bacteria by using such catheters is still discussed [43]. Lang et al. recently reported a further reduction in infection rates from 6 to 0.9% when using antimicrobial-impregnated catheters, placed in the OR combined with postoperative care on the ICU [34]. Nevertheless, it remained unclear whether or not the location for EVD placement is relevant. With that regard, it has to be mentioned that Ngo et al. reported an infection rate of 9.4% in a series of 66 patients independent if the EVD was placed in the OR or in the ICU [1].

Another question is whether routine administration of prophylactic antibiotics is necessary or not. Topjian et al. reported an infection rate of 6% when using both prophylactic antibiotics and antimicrobial-impregnated catheters [18] but they had a smaller number of patients with hemorrhages as compared to our cohort and to other series [1, 34, 43]. Children with hemorrhages, in particular those with intraventricular bleeding, are not only at higher risk to develop any EVD-related complications but also EVD-related infections requiring longer drainage periods. Also, the presence of blood in the CSF may ad as a “culture medium” for microorganisms [16, 39, 43].

Long tunneled EVDs to the chest wall or to the abdomen may reduce EVD-related infection rates further. Collins et al. had an infection rate of only 2.8% in a series of 181 pediatric patients using such techniques [33]. In another publication by Saenz et al., the group of patients with long-tunneled EVDs after explantation of an infected shunt had an infection rate of 3% as compared to 22.4% when using short-tunneled EVDs [17].

Conversion rates of EVDs into a permanent shunt system clearly depend on the primary cause for EVD placement. As expected, and as confirmed in our study, almost all patients with an infected shunt system requiring an EVD will stay shunt-dependent, thereafter. Few exemptions include those patients who had a shunt system for occlusive hydrocephalus and who may benefit then from third ventriculostomy [44]. The overall shunt conversion rates of 51.6% in our series is comparable to the series of Zhang et al. reporting a conversion rate of 64.5% [45]. When considering only the tumor and the hemorrhage groups, the conversion rates of 27.7% and 32% corresponded well with the data reported by Walker et al. indicating a rate of 25% in 180 children [2]. Recently, Krause et al. noted in a series of 36 children that preoperative EVD placement appeared to harbor a threefold relative risk for requiring subsequent permanent CSF diversion in children above 2 years [21]. This finding was supported also by another retrospective cohort study [22]. Concerning these results, the indication for placement of an EVD prior to tumor resection in the posterior fossa should be well considered, and if dispensable, it might obviate the need for a permanent shunt system. If an EVD is indicated, the drainage time should be reduced to a minimum not only to avoid EVD-related infection but probably also to lower the shunt conversion rate. The latter is exemplified by a recent publication by Zhang et al. on a series of 120 children with a medulloblastoma in whom rapid weaning of the EVD lowered postoperative shunt dependence to 18.3% [20]. Whether or not EVDs should be placed in patients with tumors in the fourth ventricle who undergo surgery in the semi-sitting position needs further consideration.

## Conclusions

While EVD-placement in children overall is safe and efficacious, more measures need to be explored to reduce both complication rates and permanent shunt dependency. At the time being, many questions remain open, and carefully planned multicenter studies are needed controlling for multivariate aspects underlying the variable complication rates—preferably in the frame of a randomized protocol despite of the difficulties implementing such study designs in the pediatric population. However, before such studies are being launched, we need clear definitions how we define “misplacement,” “infection,” and “postoperative care.” It is also relevant to consider different age groups (such as pre-term, newborns, infants, and adolescents) and specific settings with regard to local resources and regulations.

## Data Availability

No datasets were generated or analyzed during the current study.
